# Targeting Illness Perception Through TIP: Improving Depression Treatment Engagement in Late Life

**DOI:** 10.1093/geroni/igaf122.4205

**Published:** 2025-12-31

**Authors:** Rebecca Kiflom, Clare Culver, Jialin Wu, Isabel Rollandi, Helen Kales, Jo Anne Sirey

**Affiliations:** Weill Cornell Medical College, NEW YORK, New York, United States; Weill Cornell Medical College, New York City, New York, United States; Weill Cornell Medical College, New York, New York, United States; Weill Cornell Medicine, New York City, New York, United States; UC Davis Health, Sacramento, California, United States; Weill Cornell Medical College, New York City, New York, United States

## Abstract

Depression remains one of the most prevalent psychiatric disorders among older adults, contributing significantly to disability, mortality, and reduced quality of life. Despite the established effectiveness of antidepressants, adherence to antidepressant treatment remains a challenge. Illness perception, an individuals’ beliefs about the cause, timeline, and controllability of their illness, has been linked to treatment engagement across chronic health conditions but is understudied in late-life depression. This study examines whether the Treatment Initiation and Participation (TIP) intervention improved illness perception among 231 adults (>60) with major depression who were newly prescribed antidepressants at primary care clinics in Michigan and New York City. Participants were recruited within 10 days of receiving a new antidepressant prescription and randomized to either TIP or treatment as usual (control). The TIP intervention included three structured, 30-minute sessions over six weeks, during which participants explored barriers to care, discussed misconceptions about depression, addressed stigma, and developed personalized adherence strategies. Illness perception was assessed using the Brief Illness Perception Questionnaire (BIP) at baseline and again at 24 weeks. Antidepressant adherence was tracked at 6 and 12 weeks using self-report and chart verification. A mixed-effects model demonstrated TIP participants had a significantly greater average reduction in BIP scores from baseline to 24 weeks (-17) compared to the control group (-11), p<.05. These findings suggest that targeting illness perception early during antidepressant treatment may enhance understanding of depression and support sustained engagement with care. Interventions like TIP can play a role in correcting misbeliefs about depression and improving long-term adherence.

